# Supplementary material for: A catalogue of omics biological ageing clocks reveals substantial commonality and associations with disease risk
Source: Aging (Albany NY). 2022 Jan 24;14(2):623–59. doi: 10.18632/aging.203847 (PMC8833109; doi:10.18632/aging.203847)
Supplement: Supplementary Note [file aging-14-203847-s001.pdf]

SUPPLEMENTARY NOTE

Replication of omics ageing clocks trained in ORCADES in independent populations

We found clocks built using the subsets of PEA proteomics measures available in our validation cohorts correlating with chronAge nearly as highly in Croatia-Vis (r=0.89) and EBB (r=0.91) as in the ORCADES testing sample (r=0.91 and r=0.93) (Supplementary Figure 3). Similarly, both of our own DNAme Hannum and Horvath CpG based clocks achieved comparable correlations between OCA and chronAge in EBB (Hannum: r=0.98, Horvath: r=0.97) and GS:SHFS (DNAme Hannum CpGs: r=0.96, DNAme Horvath CpGs: r=0.93) as in the ORCADES testing sample (DNAme Hannum CpGs: r=0.96, DNAme Horvath CpGs: r=0.93). Our UPLC IgG glycomics and Clinomics OCA were still correlated with chronAge in independent cohorts (UPLC IgG glycomics: r=0.62 Croatia-Vis, r=0.61 Croatia-Korcula, Clinomics: r=0.56 UKBB) but less than in the ORCADES testing sample (UPLC IgG glycomics: r=0.74, Clinomics: r=0.80). There was correlation between NMR metabolomics estimated age and chronAge in Croatia-Korcula, r=0.55 compared to r=0.73 in ORCADES however only a correlation of r=0.26 in EBB. Similarly, we found that the DEXA estimated age in UKBB correlated substantially lower with chronAge than in ORCADES (UKBB: r=0.30, ORCADES: r=0.66).

To assess whether the poor correlation of DEXA OCA and chronAge in UKBB was due to the difference in the ranges of chronAge of individuals in ORCADES compared to the UKBB we also compared a clock that was evaluated in ORCADES individuals between 40-75 (the recruiting age range of UKBB, compared to the 16-100 in the full ORCADES dataset). Despite the DEXA OCA having a lower correlation with chronAge in the age restricted ORCADES sample, r=0.60 compared with r=0.66 in the full age range sample, it is still drastically higher than the r=0.30 found in UKBB.

Overlapping and unique variance in chronAge explained across omics clocks

Interestingly, the proportion of unique variance in chronAge explained by each OCA does not entirely mirror the univariate R^2 (black dots) (Supplementary Figure 5B). It is important to note that the similarity between assays likely influences the proportion of unique variance in chronAge explained (at its most extreme, were a clock duplicated, it would explain no unique variance). This may explain why NMR metabolomics, MetaboAge and MS complex lipidomics clocks have some of the lowest proportions of unique variance explained, despite NMR metabolomics and MS complex lipidomics having an R^2 higher than DEXA OCA and comparable to Clinomics. Interestingly, the DEXA and MS fatty acids lipidomics OCAs explain more unique variance than several clocks with higher univariate R^2. MetaboAge stands out with low unique variance paired with the lowest univariate R^2 in chronAge.

Pairwise clock comparisons of variance explained in chronAge

Partly to consider the effect of two similar clocks affecting the unique variance explained, we performed pairwise comparisons, the unique variance in chronAge explained by each clock in the comparison was again calculated as the squared part correlation while controlling for the other clock in the pair (Supplementary Table 6). The overlap indicated is therefore the proportion of variance in chronAge explained by both clocks in the pair. Reiterating the results in Supplementary Figures 5A, 6 shows that for 10 out of 14 clocks the mean percentage of variance explained in chronAge by both clocks (the overlap) is greater than 40%. The MS Fatty Acids Lipidomics and DEXA clocks had lower mean overlap, 23.2% and 36.9% respectively, with MetaboAge the lowest mean overlap across clocks 3.6%. Interestingly clocks that had higher correlations between OCA and chronAge, such as PEA Proteomics and DNAme-based clocks were found to be contributing most of the additional variance in chronAge not explained by the overlap of both clocks. Conversely, the MS Fatty Acids Lipidomics clock, the clock with the second lowest correlation between OCA and chronAge appears to contribute little of the additional variance in chronAge not already explained by the other clock across all comparisons. This is even more extreme for MetaboAge, in addition to extremely low average overlap in variance explained in chronAge across clocks, the other clock in the comparison contributes the majority of the variance explained. This observation, that the comparison with MetaboAge shows the lowest overlap, is consistent across all other clocks, including NMR Metabolomics which is derived from the same omics assay.

Association of chronAge and OCAAs with health outcomes

7/7 risk factors and 43/44 disease groupings associated with chronAge in the expected positive direction, except for cortisol and FEV1 which decline with chronAge (Supplementary Figure 7A, 7B). The disease exception, J00-J06 Acute respiratory infections, was not nominally significantly different from zero ($\log_e$HR/SE -0.025/

0.017). All the risk factors, and 34 of the disease grouping associations were significant after allowing for multiple testing (passed FDR 10% risk factors and diseases considered separately, one sided test H_1:b>0). For these 34 groupings there thus was reasonable power to detect associations with chronAge and so potentially biological OCAA. 2 disease groups had fewer than 5 cases and were excluded from the subsequent analysis, to further limit the burden of multiple testing.

The effect ($\log_e$HR/SE) of one year of chronAge at outset on the first incidence of any of the diseases was 0.0492/0.00323, a doubling roughly every 14 years. This pattern was generally similar to the estimated effects for each disease individually, noting these are on the same (logistic) scale. With the largest observed differences arising from diseases with larger standard errors. However, the effect ($\log_e$HR/SE) of one year of chronAge on the risk factors varied more, although again they were on the same (standardised) scale. FEV1 and systolic BP (-0.041/0.00088 and 0.035/0.0015) were most sensitive, whilst CRP and creatinine were less sensitive (effect/SE of 1 year of chronAge on standardised trait 0.0092/0.0012 and 0.0090/0.0015 respectively) as shown in Supplementary Figure 7B, whilst standard errors of the effect sizes were generally smaller (as a proportion of the effect).

Assessment of smoking as a potential confounder

Across all the associations studied for 15 clocks against 32 diseases and 7 risk factors, we found that the IVW ratio of the estimated effect of OCAA with and without smoking fitted as a covariate were 1.012 and 1.011 respectively. Individual test p-values for the ratio of the effects not being one all exceeded 0.35. Visual analysis confirmed these results: that smoking was not a material confounder of health-OCAA associations.
