# Supplementary material for: A catalogue of omics biological ageing clocks reveals substantial commonality and associations with disease risk
Source: Aging (Albany NY). 2022 Jan 24;14(2):623–59. doi: 10.18632/aging.203847 (PMC8833109; doi:10.18632/aging.203847)
Supplement: Supplementary Figures [file aging-14-203847-s002.pdf]

SUPPLEMENTARY FIGURES

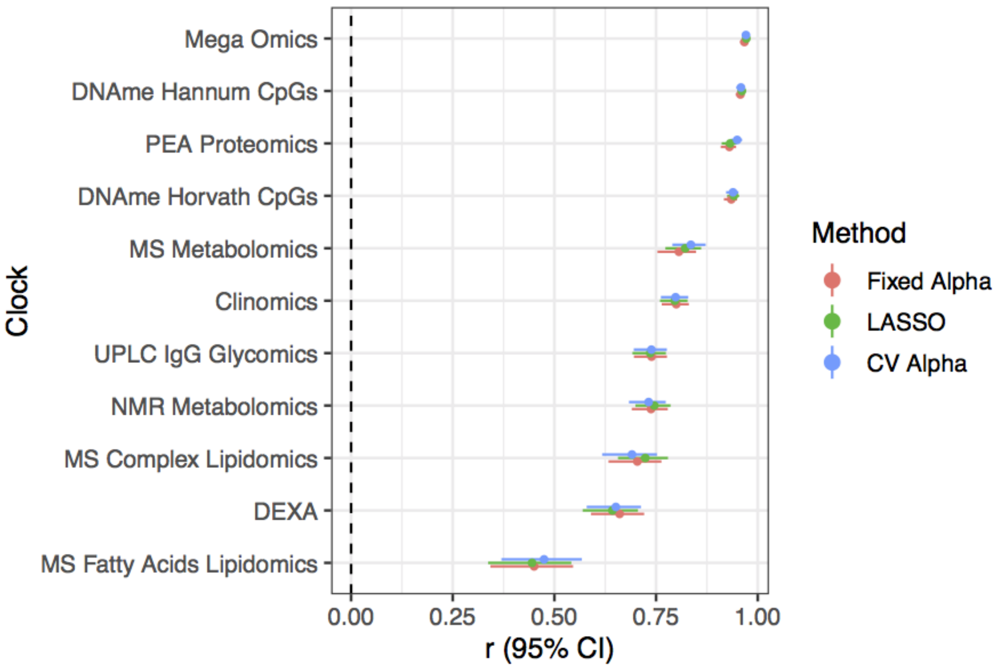

Supplementary Figure 1. Correlation of chronAge and OCA were consistent, independent of penalised regression method. Correlation (r) with 95% of confidence intervals of chronAge with omics clock estimated age (OCA) indicated on the y-axis via elastic net regression with a fixed alpha of 0.5, cross validated alpha and LASSO regression in the ORCADES testing sample.

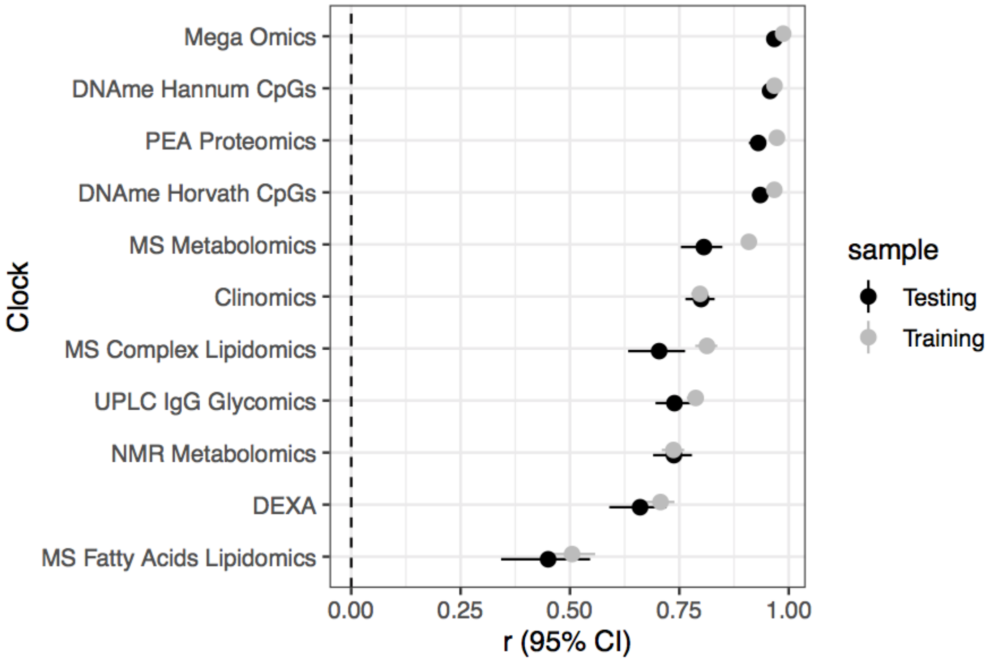

Supplementary Figure 2. Correlation of chronAge and OCA in ORCADES training and testing samples. Correlation (r) with 95% of confidence intervals of chronAge with OCA indicated on the y-axis in the ORCADES Training and Testing samples.

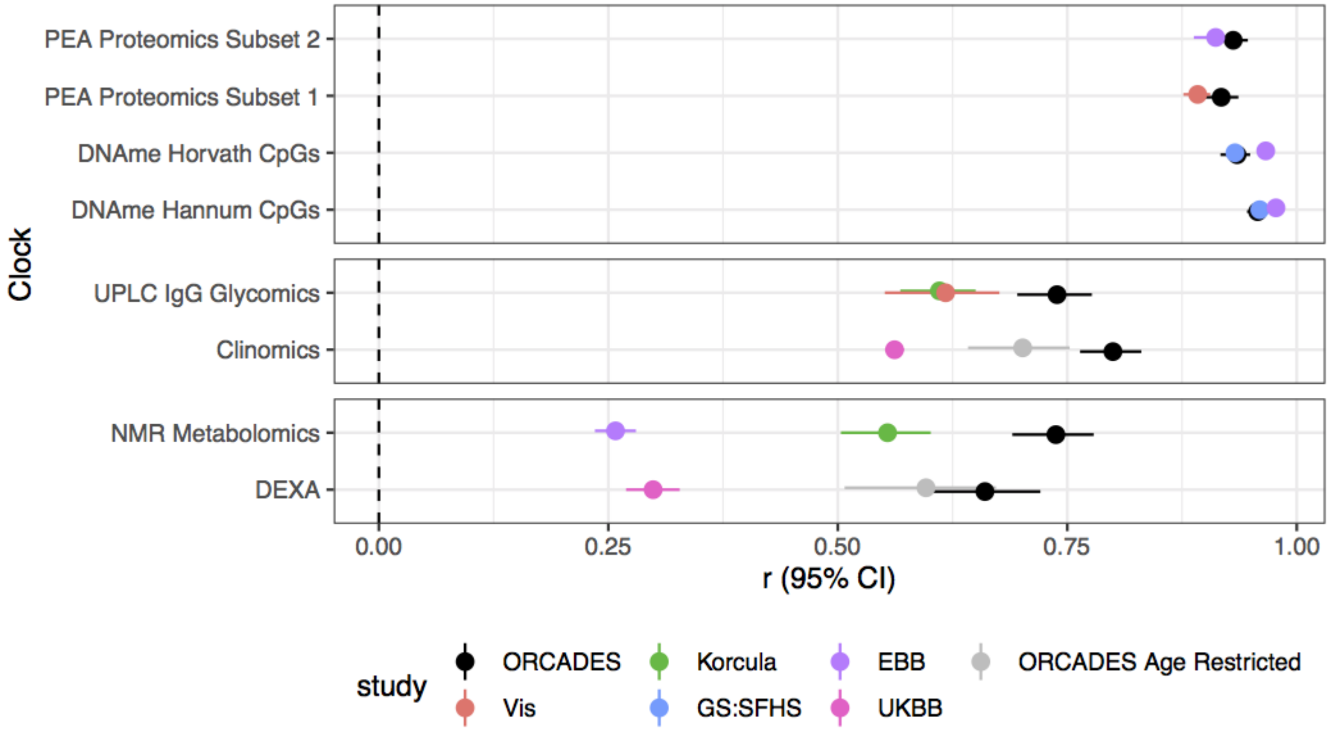

Supplementary Figure 3. Omics clocks trained in ORCADES predict chronAge in unrelated cohorts. The correlation of OCA with ChronAge (x-axis) by the specified clock (y-axis). With the correlation in the ORCADES testing sample in black and additional populations as specified. The correlation in a restricted age range (40-75) ORCADES testing sample is shown in comparisons involving the UKBB shown in grey.

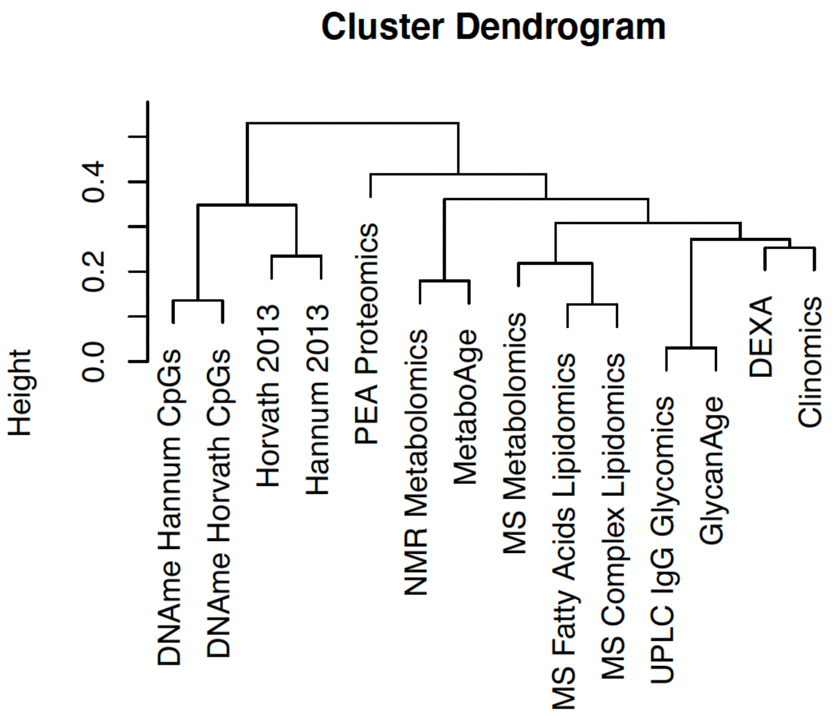

Supplementary Figure 4. Dendrogram of omics age acceleration measures. Based on hierarchical clustering of OCAA measures from each clock. Mega-Omics was excluded from this analysis as it contains predictors from multiple omics assays.

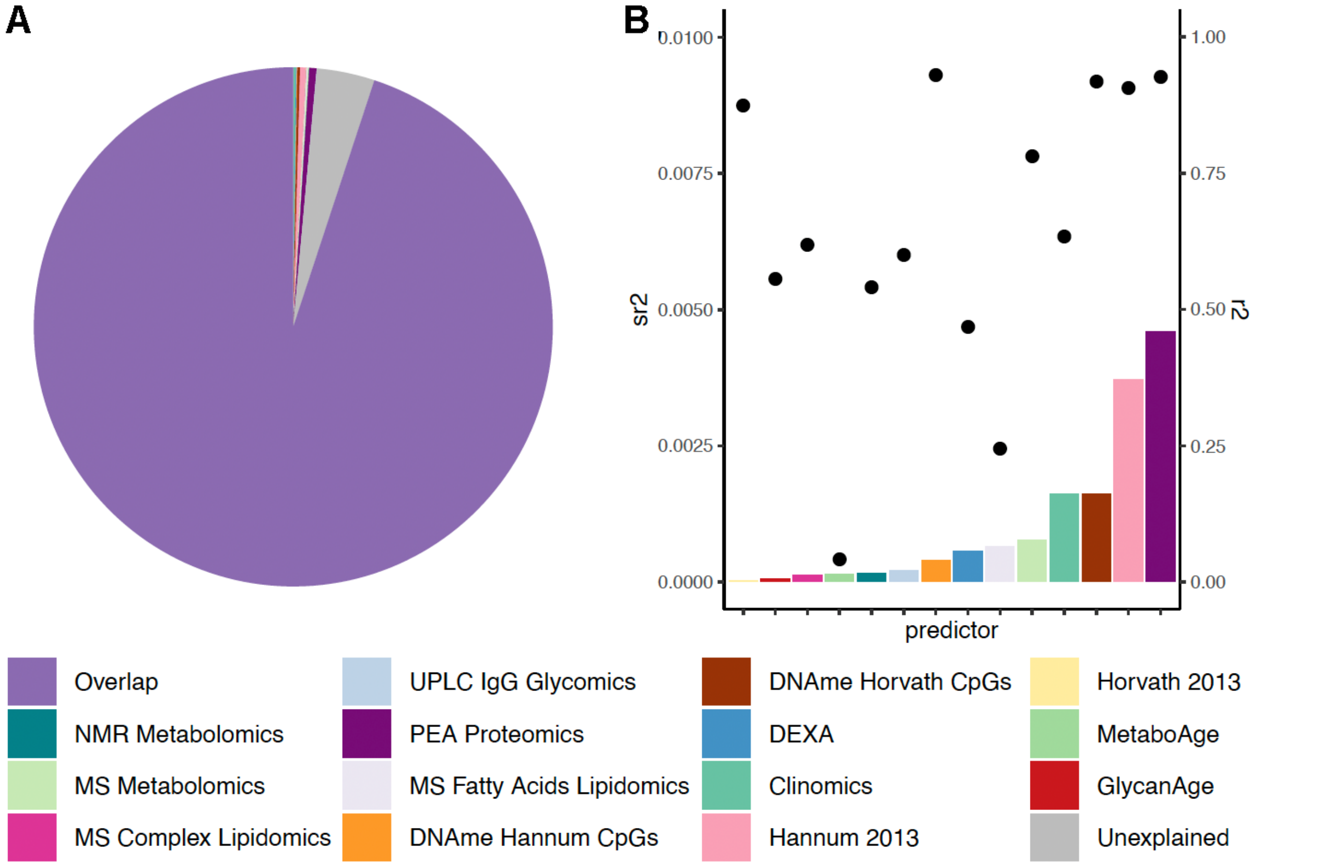

Supplementary Figure 5. Overlapping and unique variance in chronAge explained across 10 omics clocks. (**A**) Partition of variance in ChronAge explained into that explained by 2 or more clocks (overlap), that not explained by any clock (unexplained), and that explained by each of the 10 clocks uniquely. Segments coloured by component explaining the variance in chronAge. (**B**) squared part correlations (sr^2) (bars): unique variance in chronAge explained by each of the 10 clocks from Figure A on the left-hand y-axis. R^2 (points) indicate the total variance explained in chronAge by each clock (right hand y-axis).

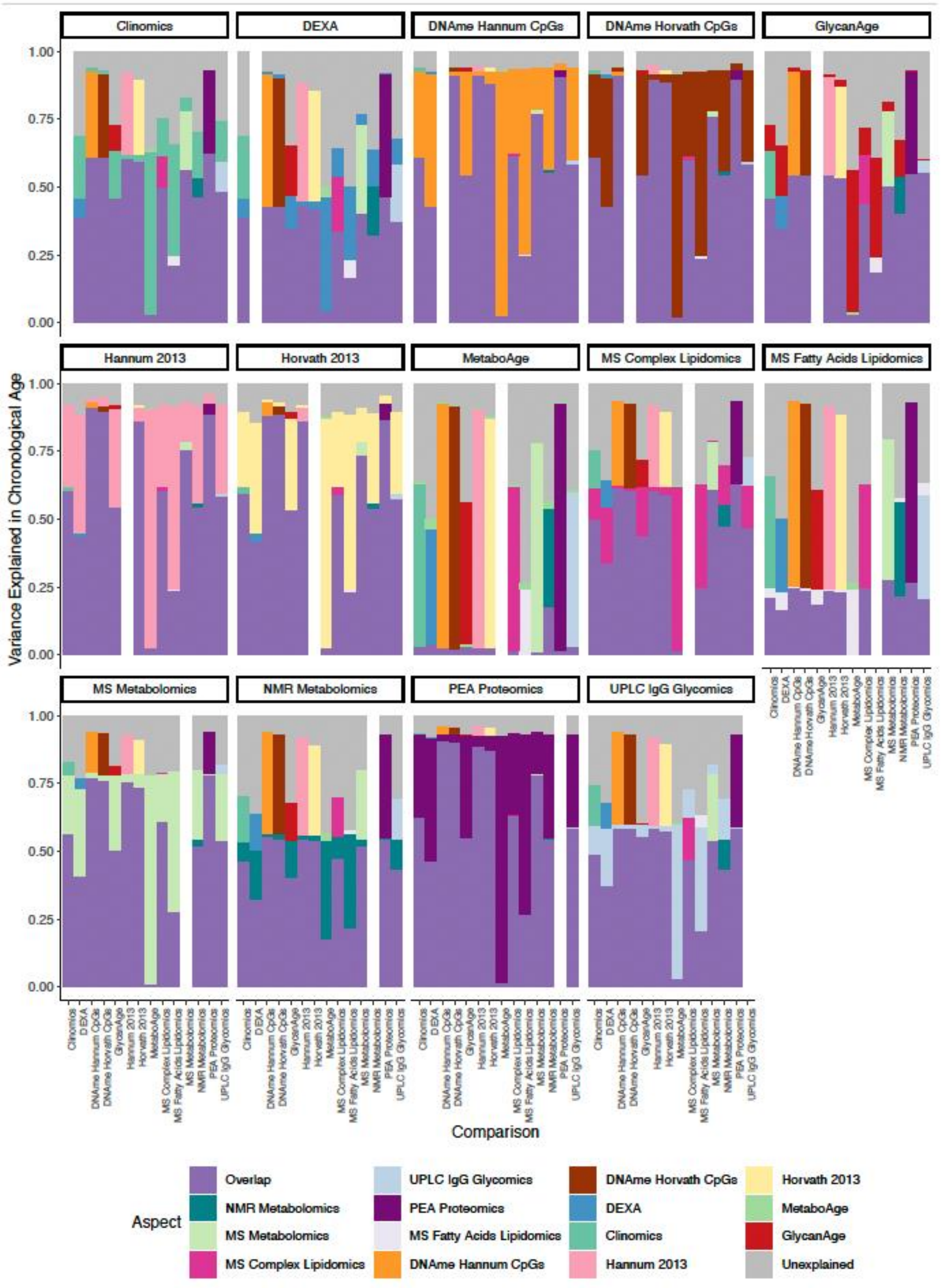

Supplementary Figure 6. Pairwise comparisons of variance explained in chronAge. Pairwise comparison of variance in chronAge explained by OCA of the pairs of clocks in ORCADES. Comparison indicated on the x-axis, with the variance in chronAge explained on the y-axis. The colour of the bar indicates the aspect explaining the variance. For each comparison the proportion of variance explained by both clocks in the comparison (Overlap), the variance that remains unexplained fitting a bivariate model (unexplained) and the unique variance in chronAge explained by each of the two clocks in the comparison.

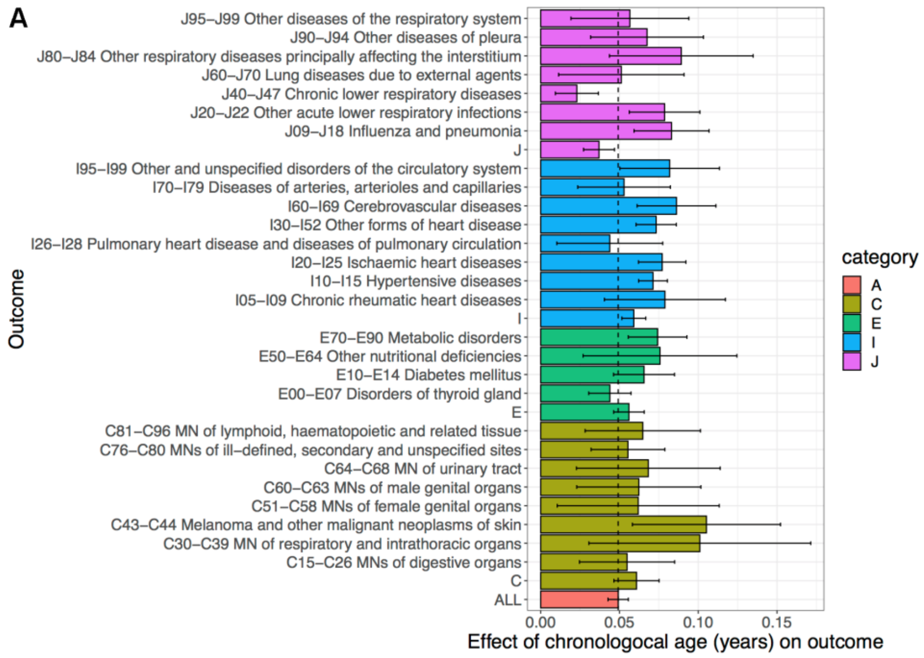

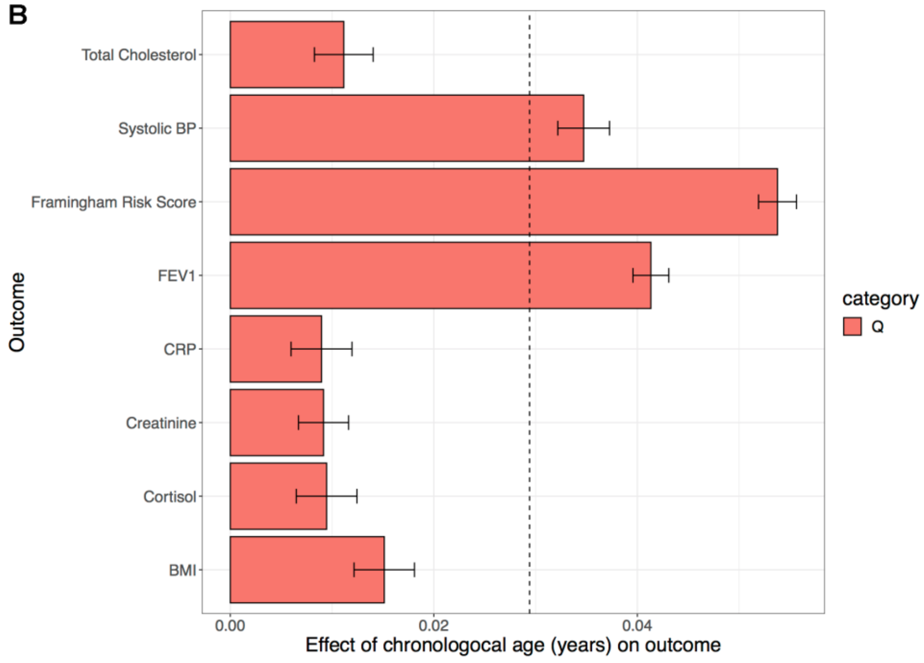

Supplementary Figure 7. Associations with disease incidence. (**A**) Associations of disease incidence with chronAge. Effect and its 95% CI: the $\log_e$HR of chronAge on the incidence of the disease since participation, using a Cox Model. ICD 10 Chapters (i.e. whole Categories) count the first occurrence (post assessment) of any disease within the letter/category/chapter (including those blocks dropped from the individual block analysis due to lack of power) as incidence. Participants prevalent at assessment (i.e. a recorded prior incidence) within any grouping at assessment were excluded from the analysis of that grouping. The dashed line represents the hazard of age on any occurrence of the disease chapters under consideration, a hazard ratio of 0.0492, representing a doubling of incidence rate every 14 years. Distinctions in observed individual effects sizes from this were (visually) judged more materially due to sampling variance than true effects, and so that single factor was chosen as our best estimate of the age effect on each disease. MNs: Malignant neoplasms. Associations are only shown for those disease groups that passed QC and were taken forward to association testing with OCAA. (**B**) The strength of associations of risk factors with chronAge varies. FEV1: Forced expiratory volume one second, CRP: C-reactive Protein, BMI: Body Mass Index. Effect: the estimated increase (and 95% CI) in standardised trait per year of chronAge using a linear model, with sex as a covariate. Traits which decrease as age increases (FEV1, cortisol) have been converted to ageing traits, by reversing their signs.

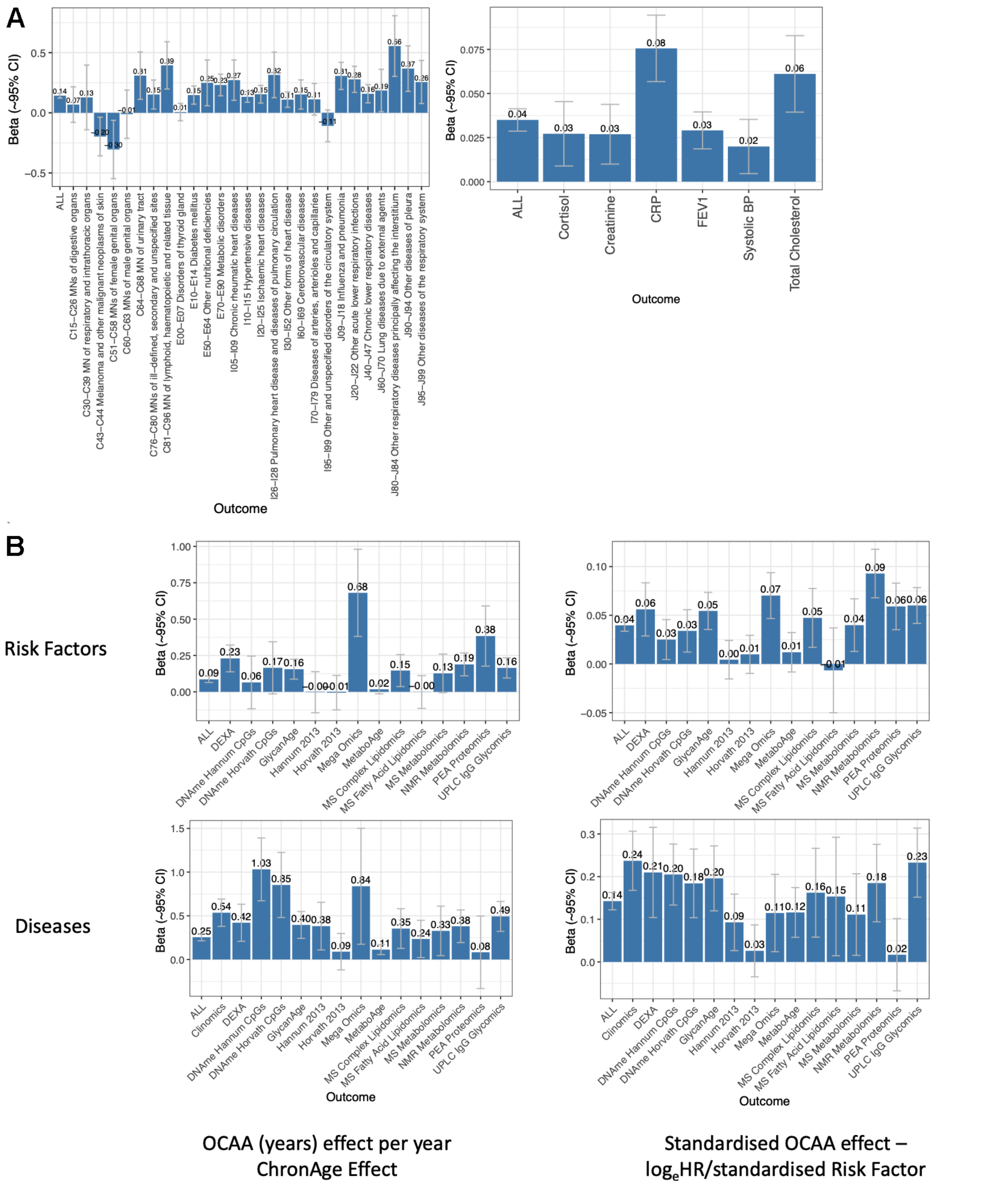

Supplementary Figure 8. (**A**) Average effect across clocks of standardised OCAA upon outcome. Beta: the observed effect of OCAAs on outcome. Beta was IVW averaged across OCAAs. SEs were calculated as the inverse root sum of the precisions (not strictly valid given correlated tests). Error bars shown are ± 2SEs. OCAA: omics clock estimated age acceleration. (**B**) Averaged effects of OCAA across diseases and risk factors. The Left-hand side shows the effect of OCAA in years per year of chronAge effect (OCAA effect divided by chronAge effect) IVW averaged across outcomes (either risk factors or diseases as specified on the y-axis). The right-hand side shows the effect of standardised OCAA (units of phenotypic standard deviation) IVW averaged across outcomes. Beta: the observed effect of OCAA on outcome. Beta was IVW averaged across outcomes. SEs were calculated as the inverse root sum of the precisions (not strictly valid given correlated tests). Error bars shown are ± 2SEs. OCAA: omics clock estimated age acceleration.

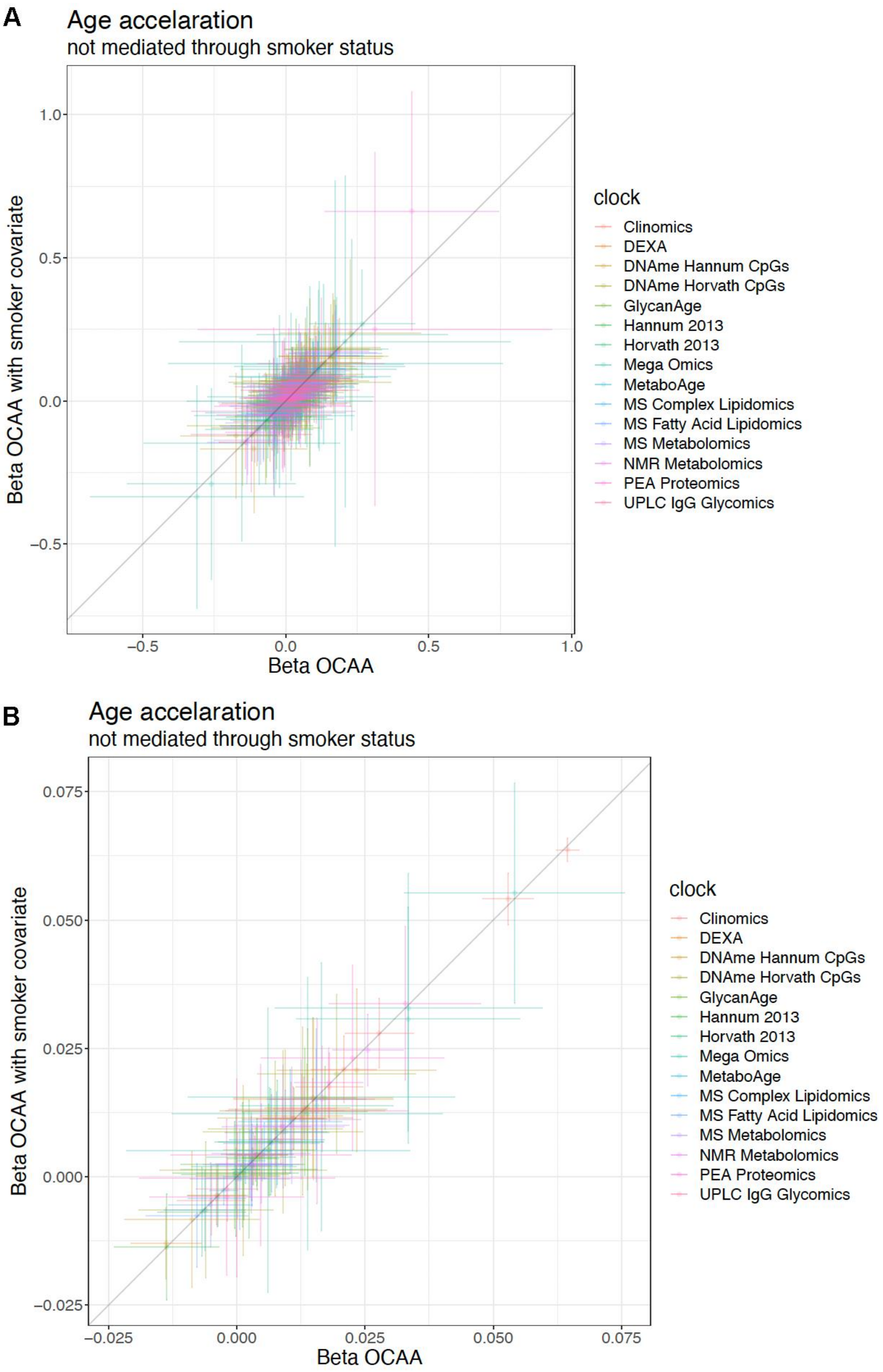

Supplementary Figure 9. Fitting smoking as a covariate does not appear to materially affect the association between OCAA and (**A**) diseases or (**B**) risk factors. Beta OCAA - the observed effect of OCAA on the outcome under the models (see main text). Beta OCAA with smoker covariate - the observed effect of OCAA on the outcome under the same model, but with smoking fitted.

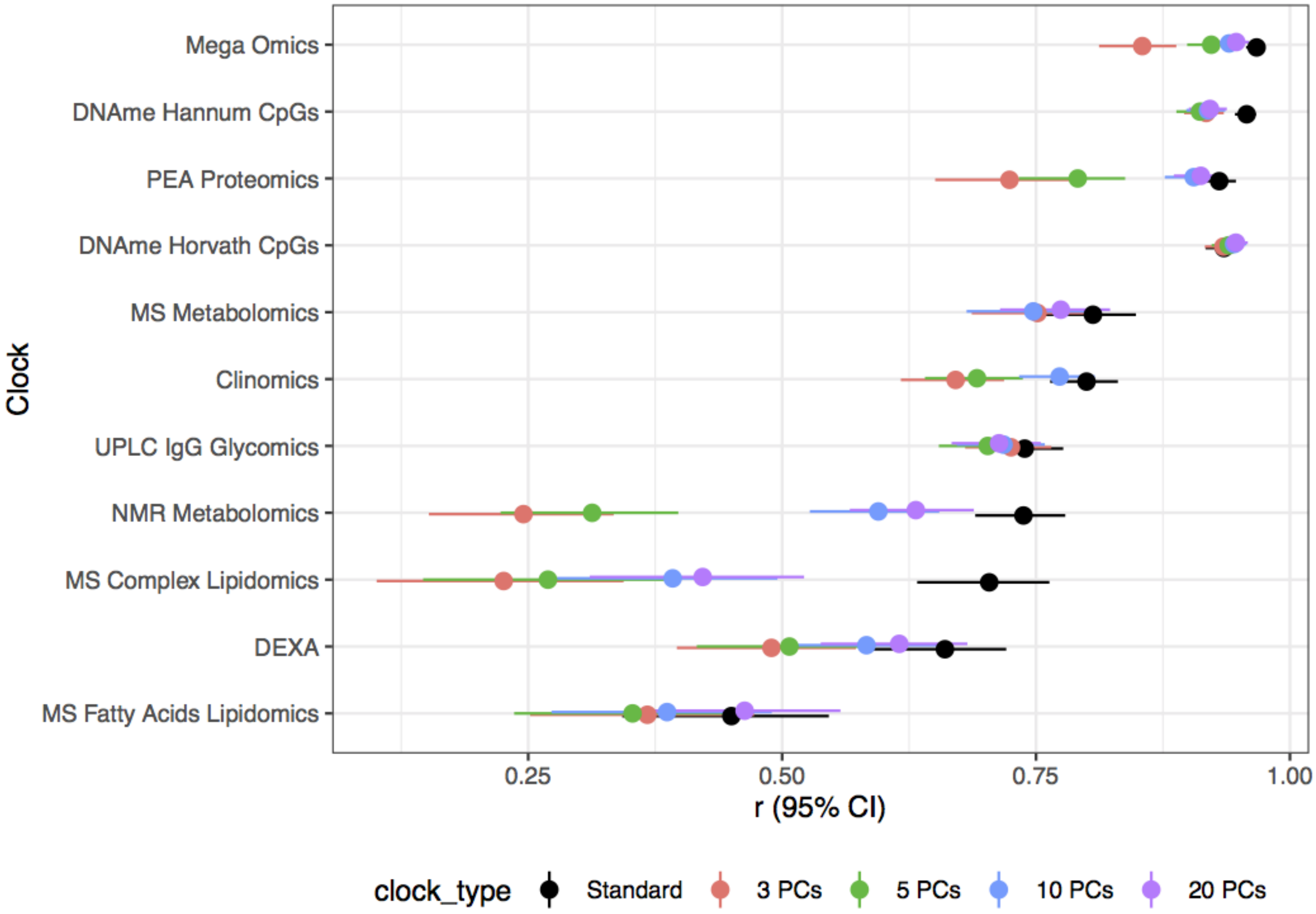

Supplementary Figure 10. Correlation of chronAge and OCA from clocks built using 3, 5, 10, 20 PCs. Correlation (r) and 95% confidence interval of chronAge and OCA indicated on the y-axis using models constructed from 3, 5, 10 and 20 principal components of the assay in the ORCADES testing sample compared to the standard clock (black).

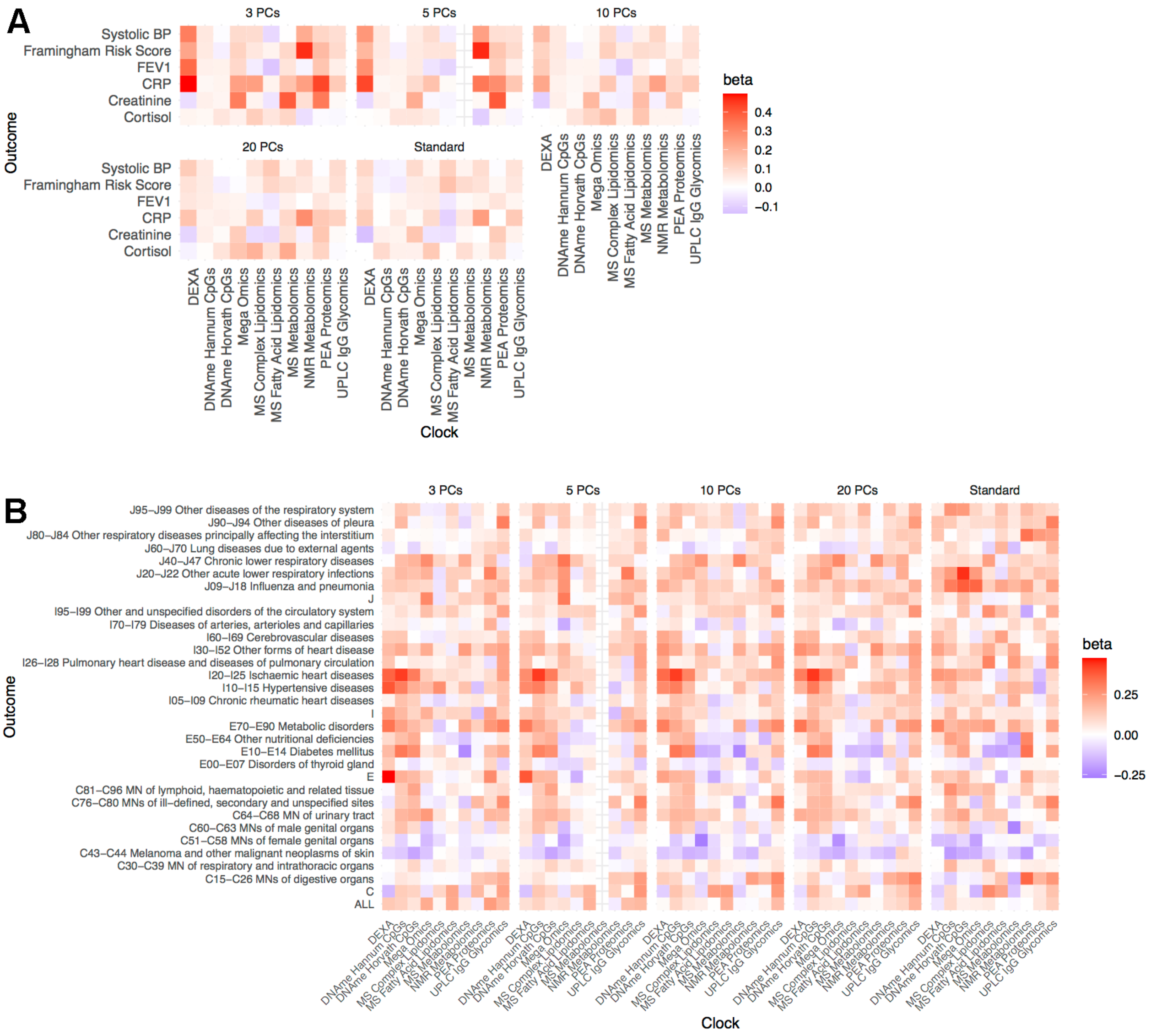

Supplementary Figure 11. (**A**) Reducing dimensionality of omics dataset used to build clocks increases the predictive ability of OCAA for risk factors. Beta: the effect of a year of standardised (within clock) OCAA on outcome (effect sizes for standardised risk factors). Estimates were shrunk using a prior to reduce the possibility that frequentist best estimate beta was predominantly a consequence of a large SE. Clock: the omics clock on which OCAA was measured. Cholesterol/BMI which showed a particularly large effect from MS Fatty Acids Lipidomics/DEXA OCAA, excluded here to aid visualisation. X PCs: the number of PCs of the omic used as predictors to create the chronAge and OCAA measures. Clinomics was excluded from this analysis as it was based on only 12 predictors. (**B**) Reducing dimensionality of omics dataset to train ChronAge makes little difference to the predictive ability of OCAA for diseases. Beta: the effect of a year of standardised (within clock) OCAA on outcome (measured in $\log_e$ hazard ratios). Estimates were shrunk using a prior to reduce the possibility that frequentist best estimate beta was predominantly a consequence of a large SE. Clock: the omics clock on which OCAA was measured. Disease group: the set of diseases (defined by ICD 10 codes) which were tested for first incidence after assessment against the clock (already prevalent cases were excluded). X PCs: the number of PCs of the omic used as predictors to create the chronAge and OCAA measures. Clinomics was excluded from this analysis as it was based on only 12 predictors.
