# Supplementary material for: A catalogue of omics biological ageing clocks reveals substantial commonality and associations with disease risk
Source: Aging (Albany NY). 2022 Jan 24;14(2):623–59. doi: 10.18632/aging.203847 (PMC8833109; doi:10.18632/aging.203847)
Supplement: Supplementary Table 1 [file aging-14-203847-s003.pdf]

Supplementary Table 1. ORCADES descriptive statistics.

Omic	N	Mean age	SD age	Min age	Max age	% Female
DEXA	1158	55.85	14.19	18.02	88	59.93
DNAme Horvath CpGs	957	52.93	15.66	17.12	100.18	55.38
MS Fatty Acids Lipidomics	952	53.41	15.49	16.84	91.47	55.78
MS Metabolomics	861	52.81	15.05	17.12	90.79	57.38
Clinomics	1815	53.35	15.03	16.5	91.47	59.56
DNAme Hannum CpGs	1033	53.43	15.68	17.12	100.18	55.86
UPLC IgG Glycomics	1937	53.13	15.29	16.5	100.18	60.51
MS Complex Lipidomics	940	53.54	15.27	17.12	91.47	55.74
NMR Metabolomics	1643	52.96	14.91	16.5	91.47	59.95
PEA Proteomics	805	52.88	15.59	17.12	91.47	54.91
Mega Omics	796	53.1	15.31	17.12	91.47	56.78
GlycanAge	1957	53.15	15.3	16.5	100.18	60.4
MetaboAge	1947	53.07	15.33	16.5	100.18	60.45
Hannum 2013	1052	53.51	15.75	17.12	100.18	55.8
Horvath 2013	1052	53.51	15.75	17.12	100.18	55.8

Omic: Omic assay.
N: number of individuals in ORCADES with the omics assay passing quality control.
Mean Age: mean chronological age at venepuncture of ORCADES subset.
SD Age: standard deviation of chronological age at venepuncture of ORCADES subset.
Minimum Age: minimum chronological age at venepuncture of ORCADES subset.
Maximum Age: maximum chronological age at venepuncture of ORCADES subset.
% Female: percentage of ORCADES subset that is female.
