# Supplementary material for: A catalogue of omics biological ageing clocks reveals substantial commonality and associations with disease risk
Source: Aging (Albany NY). 2022 Jan 24;14(2):623–59. doi: 10.18632/aging.203847 (PMC8833109; doi:10.18632/aging.203847)
Supplement: Supplementary Table 4 [file aging-14-203847-s006.pdf]

Supplementary Table 4. ICD10 definitions.

Block	Title
J95-J99	Other diseases of the respiratory system
J90-J94	Other diseases of pleura
J85-J86	Suppurative and necrotic conditions of lower respiratory tract
J80-J84	Other respiratory diseases principally affecting the interstitium
J60-J70	Lung diseases due to external agents
J40-J47	Chronic lower respiratory diseases
J30-J39	Other diseases of upper respiratory tract
J20-J22	Other acute lower respiratory infections
J09-J18	Influenza and pneumonia
J00-J06	Acute respiratory infections
I95-I99	Other and unspecified disorders of the circulatory system
I80-I89	Diseases of veins, lymphatic vessels and lymph nodes, not elsewhere classified
I70-I79	Diseases of arteries, arterioles and capillaries
I60-I69	Cerebrovascular diseases
I30-I52	Other forms of heart disease
I26-I28	Pulmonary heart disease and diseases of pulmonary circulation
I20-I25	Ischaemic heart diseases
I10-I15	Hypertensive diseases
I05-I09	Chronic rheumatic heart diseases
E70-E90	Metabolic disorders
E65-E68	Obesity and other hyperalimentation
E50-E64	Other nutritional deficiencies
E20-E35	Disorders of other endocrine glands
E15-E16	Other disorders of glucose regulation and pancreatic internal secretion
E10-E14	Diabetes mellitus
E00-E07	Disorders of thyroid gland
C81-C96	Malignant neoplasm of lymphoid, haematopoietic and related tissue
C76-C80	Malignant neoplasms of ill-defined, secondary and unspecified sites
C73-C75	Malignant neoplasms of thyroid and other endocrine glands
C69-C72	Malignant neoplasms of eye, brain and other parts of central nervous system
C64-C68	Malignant neoplasm of urinary tract
C60-C63	Malignant neoplasms of male genital organs
C51-C58	Malignant neoplasms of female genital organs
C50-C50	Malignant neoplasm of breast
C45-C49	Malignant neoplasms of mesothelial and soft tissue
C43-C44	Melanoma and other malignant neoplasms of skin
C30-C39	Malignant neoplasm of respiratory and intrathoracic organs
C15-C26	Malignant neoplasms of digestive organs
C00-C14	Malignant neoplasms of lip, oral cavity and pharynx
